# Whole exome sequencing using Ion Proton system enables reliable genetic diagnosis of inherited retinal dystrophies

**DOI:** 10.1038/srep42078

**Published:** 2017-02-09

**Authors:** Marina Riera, Rafael Navarro, Sheila Ruiz-Nogales, Pilar Méndez, Anniken Burés-Jelstrup, Borja Corcóstegui, Esther Pomares

**Affiliations:** 1 Departament de Genètica, Institut de Microcirurgia Ocular (IMO), Barcelona, Spain; 2 Departament de Retina, Institut de Microcirurgia Ocular (IMO), Barcelona, Spain

## Abstract

Inherited retinal dystrophies (IRD) comprise a wide group of clinically and genetically complex diseases that progressively affect the retina. Over recent years, the development of next-generation sequencing (NGS) methods has transformed our ability to diagnose heterogeneous diseases. In this work, we have evaluated the implementation of whole exome sequencing (WES) for the molecular diagnosis of IRD. Using Ion Proton^TM^ system, we simultaneously analyzed 212 genes that are responsible for more than 25 syndromic and non-syndromic IRD. This approach was used to evaluate 59 unrelated families, with the pathogenic variant(s) successfully identified in 71.18% of cases. Interestingly, the mutation detection rate varied substantially depending on the IRD subtype. Overall, we found 63 different mutations (21 novel) in 29 distinct genes, and performed *in vivo* functional studies to determine the deleterious impact of variants identified in *MERTK, CDH23*, and *RPGRIP1*. In addition, we provide evidences that support *CDHR1* as a gene responsible for autosomal recessive retinitis pigmentosa with early macular affectation, and present data regarding the disease mechanism of this gene. Altogether, these results demonstrate that targeted WES of all IRD genes is a reliable, hypothesis-free approach, and a cost- and time-effective strategy for the routine genetic diagnosis of retinal dystrophies.

Inherited retinal dystrophies (IRD) are a highly heterogeneous group of diseases characterized by the degeneration of photoreceptors and retinal pigment epithelium (RPE) cells. They affect approximately 1 in 3,000 people, and represent the major cause of incurable familial blindness, with more than 2 million people affected worldwide^1^. In general, IRD are classified according to the type of cells in the retina that are primarily affected —cones or rods—, the age of onset of the first symptoms, and the progression of degeneration over the years^2^. Accordingly, the spectrum of IRD comprises diseases that predominantly affect (i) the central retina, such as Stargardt disease (STGD), cone dystrophy (CD), cone–rod dystrophy (CRD), and achromatopsia (ACHR); (ii) the peripheral retina, such as retinitis pigmentosa (RP); or (iii) both, such as Leber congenital amaurosis (LCA). However, some patients do not display specific clinical representations associated with a particular IRD, but exhibit overlapping phenotypes that are consistent with more than one particular dystrophy. Moreover, intrafamilial variability and incomplete penetrance are not uncommon, so assigning a definitive and precise clinical diagnosis can be difficult. In most cases, the retina is the only affected tissue (non-syndromic forms), while in some patients, other tissues can also be involved (syndromic forms)^3^. The latter includes Usher syndrome (US), Bardet-Biedl syndrome (BBS), and Joubert syndrome (JS), among others. In some families, discrimination between a non-syndromic or syndromic IRD is not always simple, as the existence of affected tissues other than the retina can be coincidental^4^. In this regard, genetic analysis becomes essential, as it is the only current tool that has the potential to provide a reliable and conclusive diagnosis. Nevertheless, providing a molecular diagnosis for these pathologies can be challenging due to the large number of candidate genes (>200) and mutations (>4,000) described, the absence of major genes or mutations, multiple inheritance patterns (autosomal dominant, autosomal recessive, and X-linked), the possible existence of modifier alleles, and the presence of non-causative variants^5^.

Previously, conventional genetic diagnosis of IRD patients was based on the use of arrayed primer extension reactions (APEX) and Sanger sequencing method, which allow for the examination of known mutations or specific exons and gene targets^6^. However, these techniques guarantee the identification of the causative mutations in only 10–20% of cases^7^. More recently, next-generation sequencing (NGS) technologies have revolutionized genetic diagnosis of IRD. These methods facilitate the simultaneous screening of a large number of genes, are at least 1,000 times faster than conventional sequencing, and are much less expensive per sequence^8^. Within this context, some authors have opted to develop their own disease-specific gene panels that include a list of genes responsible for one particular IRD subtype^9^,^10^,^11^,^12^,^13^,^14^, but they can only be applied when a clinical interpretation can be made with relative assurance^15^.

Different studies suggest that 35% of IRD cases remain unsolved at the genetic level^2^; however, it is thought that the most common IRD genes have already been identified. Therefore, undiagnosed patients may carry mutations either in syndromic genes (usually not analyzed in non-syndromic patients), in genes associated with other IRD subtypes (not included in disease-specific panels), or in as-yet-unknown IRD genes^4^,^16^. This fact suggests that the best approach for the genetic diagnosis of these pathologies may be the simultaneous analysis of known genes responsible for both syndromic and non-syndromic forms of all IRD types. This could be through targeted/custom non-disease-specific panels or through more broad-based NGS strategies such as whole exome sequencing (WES) or whole genome sequencing (WGS). Within this context, new associations between already-known IRD genes and particular phenotypes would be established. Indeed, during the last few years, many IRD genes have been reclassified using these approaches^12^,^17^,^18^,^19^,^20^,^21^. In the present study, we used WES to examine 212 genes responsible for more than 25 syndromic and non-syndromic retinal dystrophies in a cohort of 59 unrelated families affected by a wide range of IRD phenotypes.

## Results

### Clinical diagnosis of IRD patients

Almost 89% of the cases included in our cohort had a well-defined clinical diagnosis (52/59). Most of the phenotypes were non-syndromic (53/59), with RP being the most common of the IRD (26 isolated cases and six US cases), followed by CD and STGD (six and five cases, respectively) (Table 1). In seven cases (11.86%), a specific clinical diagnosis could not be assigned, as the displayed phenotype was compatible with two, or even three, different retinal dystrophies (unclear phenotype).

### WES approach for the genetic diagnosis of IRD patients

Ion Ampliseq^TM^ Exome technology and Ion Proton^TM^ platform allowed for the capturing, amplification and sequencing of more than 97% of the coding regions of >19,000 genes. The generated data was filtered to obtain coverage information and variants of 212 genes included in our IRD panel (the full list of genes is given in Supplementary Table S1). In particular, 5,294 amplicons covered 98.4% of the coding regions and flanking exon/intron boundaries (around 50 bp) of the IRD genes. After the analysis, 96.9% of target regions had a coverage of >20x, 90% had >40x, 56.4% had >100x, and 12.8% had >200x. Only 3% of the amplicons had coverage of <20x (Supplementary Fig. S1).

**Figure 1:**
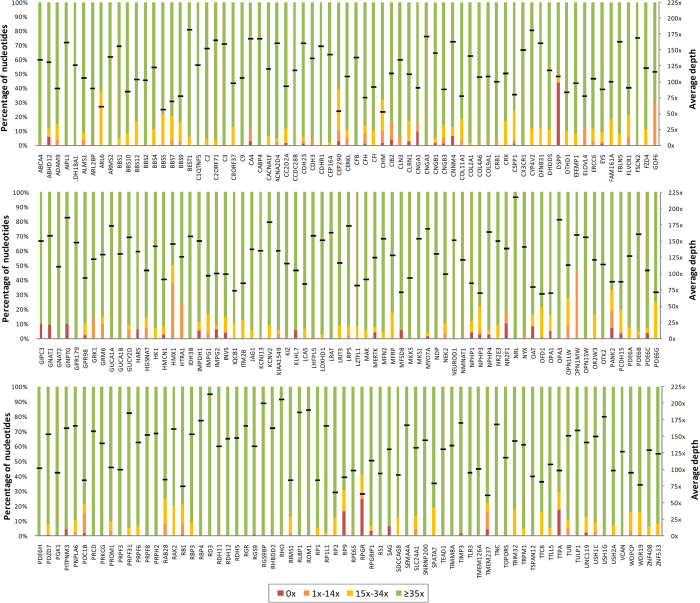
Coverage statistics of coding regions of genes included in the panel. The percentage of nucleotides with 0x, 1x–14x, 15x–34x, or ≥35x depth coverage per gene is shown. Black lines represent the average depth in each case.

On average, depth coverage of IRD genes was 121x, with values that ranged from 52x (*CHM*) to 217x (*NRL*). Most of the genes (73.5%, 147/212) displayed an average depth of >100x. A schematic representation of the percentage of nucleotides covered at different depth ranges, as well as the mean value for each gene, is shown in Fig. 1.

**Figure 2:**
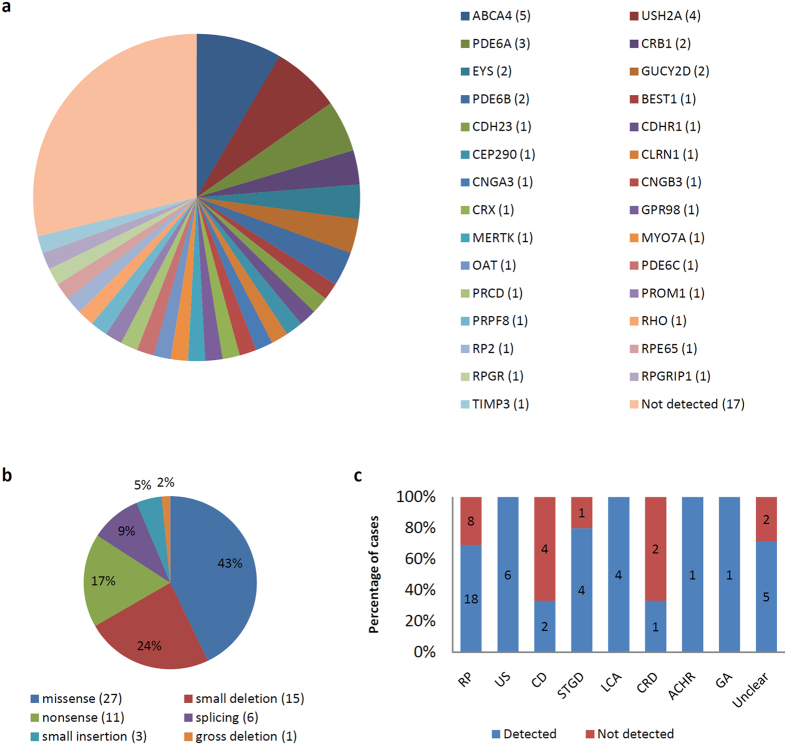
Mutations identified in a cohort of 59 IRD families using a targeted WES strategy. (**a**) Distribution and frequencies of IRD genes. *ABCA4, USH2A* and *PDE6A* were the most prevalent genes. (**b**) Types of mutations identified and their frequencies. (**c**) Comparison of the mutation detection rate of different IRD subtypes. Abbreviations: ACHR, achromatopsia; CD, cone dystrophy; CRD, cone–rod dystrophy; GA, gyrate atrophy; LCA, Leber congenital amaurosis; RP, retinitis pigmentosa; STGD, Stargardt disease; US, Usher syndrome.

For each sample, a mean of 1,050 variants were identified in the 212 genes, including single nucleotide variants and small indels. After discarding common polymorphisms (MAF > 0.01), putative false-positives (depth < 15x), and non-deleterious variants (synonymous substitutions, UTR variants), a mean of 14 variants remained, for which we used different prediction tools to evaluate their putative pathogenic impact. In addition, cosegregation studies were performed when relatives’ samples were available. In the present study, a total of 63 different causative mutations were identified in 29 distinct genes (Table 2). *ABCA4, USH2A*, and *PDE6A* were the most frequent causative genes in our cohort (the frequency distribution of each gene is shown in Fig. 2a). Of the 63 mutations, 21 were identified for the first time (33.3%). A deleterious function of new missense variants was attributed when at least three of the four prediction programs assigned a damaging or disease-causing effect (see Supplementary Table S2). In family Fi15/09, the missense variant c.3G > A affected the first methionine of *PROM1*; therefore, it was assumed to be deleterious, regardless of the bioinformatic predictions. The pathogenic effect of novel variants located in introns was estimated using five splice site predictors. Only those variants estimated to weak canonical splice sites, or those that create new donor or acceptor sites, were considered pathogenic (Supplementary Table S3). The identified mutations were mainly missense (43%), followed by small deletions (24%), nonsense (17%), splicing (9%), small insertions (5%), and gross deletions (2%) (Fig. 2b).

The causative mutation (or mutations) was identified in 71.18% of the families (42/59). The mutation detection rate varied considerably depending on the retinal dystrophy subtype. In some diseases, such US and LCA, it reached 100%; however, in CD and CRD, it did not exceed 35% (Fig. 2c). Regarding RP, pathogenic mutations were identified in 18/26 cases (69.2%). Interestingly, phenotypic unclear cases reached a mutation detection rate of 71.4% (5/7). Finally, the genetic diagnosis was inconclusive in 17 families, representing 28.81% of the cohort, although in four of these, we identified one putative pathogenic variant in a recessive gene (see Supplementary Table S4).

### *CDHR1* as a candidate for retinitis pigmentosa with early macular affectation

**Figure 3:**
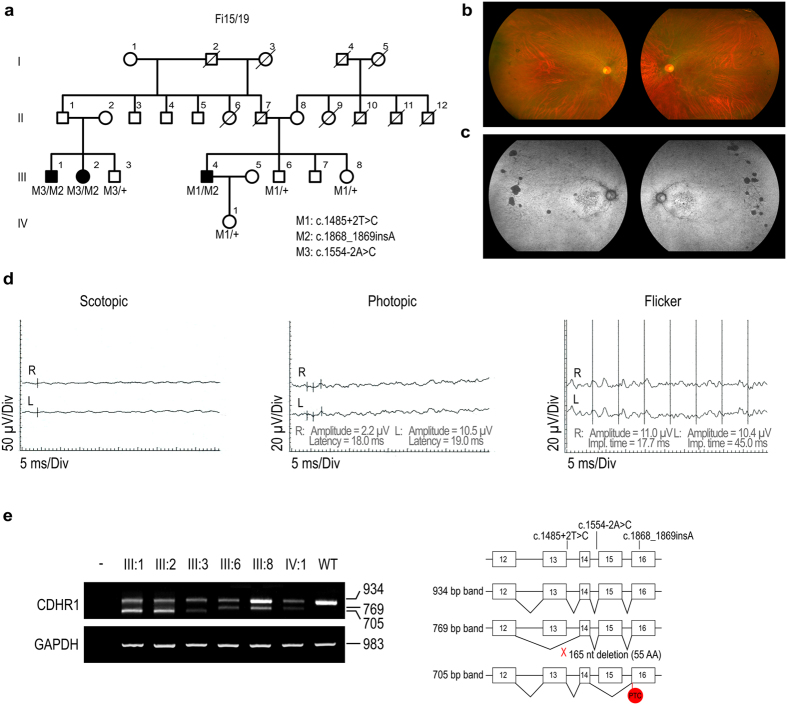
Identification of *CDHR1* mutations in a family affected by RP. (**a**) Cosegregation analysis of *CDHR1* variants identified in family Fi15/19. (**b** and **c**) Fundus eye photographs and autofluorescence images of the affected member III:2. (**d**) Electroretinographic recordings under scotopic (0 dB) and photopic (0 dB) conditions from both eyes of patient III:4. A b-wave could be detected in photopic ERG (amplitude and latency values are shown). Photopic 30-Hz flicker (0 dB) was also recorded. (**e**) RT-PCR analysis of *CDHR1* blood mRNA of affected patients (III:1 and III:2), carriers (III:3, III:6, III:8, and IV:1), and one control individual (WT). Patients and carriers showed a dramatic decrease in the *CDHR1* normal transcript. III:1, III:2, and III:3, who carry c.1554-2A > C, also produced a lower mass band corresponding to an mRNA that skips exon 15, whereas members III:6, III:8 and IV:1, carriers of c.1485 + 2T > C, presented a 769 bp band that directly links exon 12 and 14 of the gene.

Spanish family Fi15/19 contained three affected members from two different branches (two siblings, III:1 and III:2, and their cousin, III:4) who developed progressive nyctalopia and a reduction in peripheral visual field during the second decade of life (Fig. 3a). Patient III:4 presented a best-corrected visual acuity (BCVA) of 20/32 and 20/40 at the ages of 25 and 35 years, respectively. BCVA decreased rapidly between the fourth and fifth decade of life, progressing to light perception at the age of 45. Fundus examination of the affected members of the family revealed typical signs of RP, including marked attenuation of the retinal blood vessels, waxy pallor of the optic discs, and pigmented bone spicules in the periphery (Fig. 3b). Retinal autofluorescence showed diffuse RPE disturbances, both in the periphery and the macula. The macula showed a mottled hypoautofluorescence, similar to that seen in the very early stages of CRD, but without the characteristic RPE atrophy seen in the later stages (Fig. 3c). Electroretinography (ERG) showed abolished responses in scotopic conditions. The a-wave was also undetectable in photopic ERG, though there was a clearly reduced, but detectable, b-wave (Fig. 3d). The clinical and electrophysiological findings showed some overlapping traits between CRD and RP. However, the initial symptoms were clearly dominated by nyctalopia and peripheral visual field loss, while central vision decreased later, although to a much greater extent than found for most cases of RP. Altogether, these findings support a diagnosis of RP with early macular degeneration for this particular family.

Genetic analysis using the IRD panel showed that patient III:4 carried a frameshift mutation (c.1868_1869insA, p.Asn623Lysfs*53) in compound heterozygosity with a splicing variant (c.1485 + 2T > C) in the *CDHR1* gene. The first variant was considered to be new as it was not found in any public or private database, whereas the splicing variant was previously described in patients affected with a diffuse retinal dystrophy involving both cones and rods^22^. Cosegregation analysis of family Fi15/19 revealed that the variants came from different alleles, that no healthy sibling carried both variants, and that affected members III:1 and III:2 only carried the c.1868_1869insA variant. At this point, all coding regions of *CDHR1* were analyzed by Sanger sequencing in patient III:2, finding a previously unknown putative pathogenic splicing variant, c.1554-2A > C (Fig. 3a). All the splicing predictors defined this new variant as clearly pathogenic (Supplementary Table S3).

In order to confirm the pathogenicity of these three *CDHR1* variants *in vivo*, RT-PCR analysis was performed on RNA blood samples from two affected members (III:1 and III:2), four carriers (III:3, III:6, III:8, and IV:1), and one non-related control individual (wild-type [WT]). The results revealed that affected patients and most carriers presented lower levels of the *CDHR1* canonical isoform compared with the WT (934 bp band in Fig. 3e). Interestingly, family members III:1, III:2 and III:3 (carriers of the c.1554-2A > C variant) also showed a 705 bp band that skipped exon 15 of the gene. This generated transcript created several premature termination codons (PTC) and apparently did not undergo nonsense-mediated mRNA decay (NMD), maybe due to the position of the first PTC, located in the second-to-last exon. On the other hand, members III:6, III:8 and IV:1 (carriers of the c.1485 + 2T > C variant) produced a 769 bp band, which directly linked exons 12 and 14, and generated an in-frame transcript that likely translates to a CDHR1 protein that lacks 55 amino acids of two cadherin domains. Concerning the frameshift mutation (c.1868_1869insA), it was detected at the cDNA level of the carriers, suggesting that this variant also avoids NMD.

### Novel functional studies of IRD mutations

#### Retinitis pigmentosa

**Figure 4:**
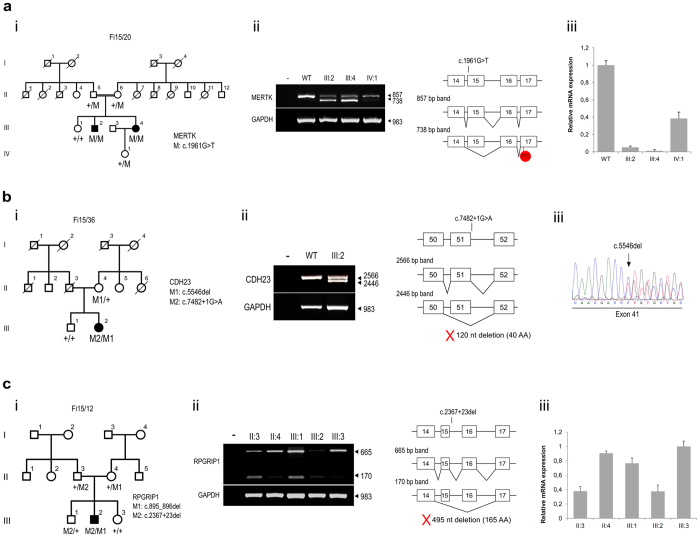
*In vivo* functional studies of variants identified in *MERTK, CDH23*, and *RPGRIP1* genes. (**a-i**) Pedigree of consanguineous family Fi15/20, showing the cosegregation analysis of variant c.1961G > T of *MERTK*. (**a-ii**) Electrophoresis gel of the RT-PCR products obtained from affected patients (III:2 and III:4), carrier (IV:1), and WT blood samples. Patients produced high levels of an aberrantly spliced transcript, whereas the WT produced only the expected 857 bp band. (**a-iii**) Quantitative analysis of *MERTK* levels by real-time RT-PCR. The WT sample was set at 100%. (**b-i**) Cosegregation analysis of the two new *CDH23* variants identified in family Fi15/36. (**b-ii**) RT-PCR assay using blood samples from the affected patient (III:2) and an unrelated WT individual. Two different bands were obtained in the patient, the expected one and another skipping exon 51. (**b-iii**) Chromatogram of the 2,566 bp band obtained from cDNA of patient III:2. (**c-i**) Fi15/12 family pedigree and *RPGRIP1* variants cosegregation. (**c-ii**) RT-PCR analysis revealed intrafamilial differences in the expression of *RPGRIP1* gene. (**c-iii**) Quantification of *RPGRIP1* canonical transcript by real-time RT-PCR. The noncarrier family member III:3 was used as a control, and her sample was set at 100%.

Pedigree Fi15/20 is a Spanish consanguineous family with two siblings severely affected by RP. The analysis of the IRD panel in patient III:2 revealed a novel homozygous missense mutation in *MERTK*, c.1961G > T (p.Gly654Val). This variant cosegregated with the disease (Fig. 4a–i) and was predicted as deleterious by different missense prediction algorithms (Supplementary Table S2). Interestingly, the variant affected the first nucleotide of exon 15 (a highly conserved guanine), which highlighted a putative effect on the splicing mechanism. In accordance with the splicing predictors, this variant abolishes the recognition of the intron 14 acceptor site (Supplementary Table S3). To assess whether the c.1961G > T mutation resulted in an altered splicing pattern, we performed a comparative RT-PCR analysis of *MERTK* in RNA from white blood cells of the affected siblings (III:2 and III:4), one carrier (IV:1), and one non-related control individual (WT), using primers located within exons 11 and 18 of the gene (see Supplementary Table S5 for primer sequences). The WT sample produced a single 857 bp band, as was expected from analysis of a correctly spliced transcript, whereas both patients and the carrier showed not only the WT band, but also another lower band of 738 bp, which lacked exon 15 and directly fused exons 14 and 16 (Fig. 4a–ii). This aberrant transcript contained PTC and was found abundantly in the patients, indicating that it probably avoids the NMD degradation mechanism. The level of WT transcript was quantified in each sample using real-time RT-PCR, showing that the carrier individual produced around 38% WT transcript, whereas the patients only expressed 5–10%, compared with the control sample (Fig. 4a–iii). However, it is worth mentioning that this small amount of correct mRNA still carried the missense mutation, which is predicted to be deleterious.

#### Usher syndrome

The analysis of individual III:2, of the Spanish family Fi15/36, who was diagnosed with US, identified two novel mutations in *CDH23*: a deletion of a single nucleotide that creates a frameshift mutation (c.5546del, p.Pro1849Leufs*4), and a splicing variant (c.7482 + 1G > A) (Fig. 4b–i). In order to assess the effects of the two variants on *CDH23* expression, RT-PCR analysis of blood samples from the patient and a non-related WT individual was performed using primers located in exons 39 and 54. In contrast with the WT sample, two different transcripts were obtained from the affected patient: one corresponding to the WT band (2,566 bp), and another (2,446 bp) skipping exon 51 (Fig. 4b–ii). This finding suggests that the c.7482 + 1G > A splicing variant weakens the canonical donor splice site of exon/intron 51. The direct link between exons 50 and 52 generated an in-frame transcript that likely produces a CDH23 protein that lacks 40 amino acids of a highly conserved sequence of the extracellular domain. Concerning the c.5546del frameshift mutation, Sanger sequencing of the obtained WT band from the patient’s cDNA suggested that the aberrant transcript did not undergo NMD degradation, as the chromatogram showed double peaks starting from the variant position (Fig. 4b–iii).

#### Leber congenital amaurosis

Family Fi15/12, which contained one male affected with LCA, was previously genetically analyzed by de Castro-Miro *et al*. using a cosegregation chip based on SNP genotyping, followed by Sanger sequencing of the candidate genes^23^. This analysis identified two new variants in *RPGRIP1*, a deletion of two nucleotides that created a frameshift mutation (c.895_896del, p. Glu299Serfs*21), and an intronic variant with unknown pathogenic effect (c.2367 + 23del) (Fig. 4c–i). However, the genetic diagnosis was finally inconclusive, as the pathogenic impact of the intronic variant could not be proved. At this point, with consideration of the family’s interest in obtaining a reliable molecular diagnosis, the affected patient was analyzed using the IRD panel. Nevertheless, no clearly pathogenic mutations were detected other than the two *RPGRIP1* variants. Within this context, we aimed to evaluate the pathogenic impact of the c.2367 + 23del variant, which did not show any substantial pathogenic impact according to the *in silico* predictions (see Supplementary Table S3). The *RPGRIP1* gene displays high transcriptional complexity, with two different promoters (the canonical one and another internal promoter located before exon 10) and several alternative exons. Previous studies in animal models have revealed that many of the *RPGRIP1* isoforms are likely to have tissue-specific expression, and that both promoters are active in the retina, whereas only the one with the transcription start site in exon 10 is active in other tissues such as the lung, heart, or testis^24^,^25^. Thus, we first assessed the expression of *RPGRIP1* isoforms in human blood by using several pairs of primers, with cDNA amplification obtained when using primers located after exon 10, suggesting that the internal promoter is the only one active in this tissue. Taking into account these results, RT-PCR analysis using primers located at exons 14 and 17 was performed in the affected patient (III:2), his carrier parents (II:3 and II:4), the carrier brother (III:1) and the noncarrier sister (III:3). (see Supplementary Table S5 for primer sequences). All family members expressed two different bands, the WT band (665 bp) and a lower one (170 bp) that lacked exons 15 and 16 (Fig. 4c–ii). Intrafamilial variable expression of these two transcripts was observed. At this point, we aimed to quantify by real-time RT-PCR the amount of WT transcript in each case, using a Taqman probe located between exons 14 and 15 (Fig. 4c–iii). Interestingly, those family members who carried the intronic variant exhibited reduced expression of the WT isoform ranging from 37–75%. This suggests that the c.2367 + 23del variant strongly impairs the recognition of *RPGRIP1* canonical splice sites, favoring the production of the transcript that skips exons 15 and 16. The latter would likely generate an in-frame sequence that lacks 492 nucleotides (164 amino acids) that code for the C2 protein domain, which is responsible for RPGRIP1 interaction with other proteins in the ciliary transition zone. In this regard, several *RPGRIP1* deleterious mutations have been shown to disrupt this interaction^26^,^27^.

## Discussion

Some authors claim that custom targeted-NGS of specific panels is the best strategy for genetic screening of IRD, and that WES is useful for uncovering new candidate genes involved in these diseases only when known genes have already been ruled out^28^. In this report, we propose that WES is an effective tool not only for the identification of new genes, but also for routine IRD molecular diagnosis. Within this context, we used Ion Proton^TM^ system to sequence coding regions of > 19,000 genes and specifically analyze a panel of 212 IRD candidates in a cohort of 59 genetically and clinically unselected families. The pathogenic variants were detected in 71.18% of cases (42/59), reaching a mutation detection rate higher than most previous studies, where either custom targeted-NGS or WES was used (Table 3)^9^,^10^,^11^,^13^,^19^,^29^,^30^,^31^,^32^,^33^,^34^,^35^,^36^,^37^,^38^,^39^. The improvement displayed in our study may be explained by a number of different factors: i) more accurate clinical characterization, which assures that all patients included in the cohort show features compatible with retinal dystrophies caused by a genetic alteration; ii) superior gene panel design, ensuring inclusion of all potential candidates; iii) a more optimal data analysis pipeline (for example, 15x as the acceptable coverage threshold); and iv) better coverage of the genes of interest, especially those that contain mutational hotspots or prevalent variants.

WES offers several advantages compared to targeted-NGS, especially in genetic heterogeneous diseases such as IRD, for which novel disease genes are continually being discovered. In this regard, targeted-NGS is limited in its flexibility for including new disease candidates, whereas the WES strategy allows for rapid enlargement of the panel. Moreover, for unsolved cases, once all known genes have been ruled out by WES, the same generated data can be used for the identification of new candidates located throughout the entire exome. However, it is worth mentioning that targeted-NGS retains some advantages as it can achieve a higher depth of coverage in the regions of interest than that possible with WES^37^. In our case, the mean depth of the genes included in the panel was 121x, whereas in most targeted-NGS studies, this value ranged between 250x and 1,330x (Table 3)^9^,^10^,^13^,^19^,^31^,^36^,^37^. A greater depth of coverage not only reduces the detection of false-positives, but also allows for the analysis of copy number variations (CNV)^4^,^6^. However, in complex sequences such as exon ORF15 of *RPGR*, custom targeted-NGS does not achieve better coverage than that possible with WES. Our strategy allowed for the analysis of 76% of the whole *RPGR* gene and 41% of exon ORF15, with these values being similar to previous target-NGS studies^9^. Within this context, we were able to identify by WES a frameshift mutation in exon ORF15 in one patient of the cohort (Fi15/41).

In our study, CNV detection was not possible due to the average depth obtained; thus, we assume that gross rearrangements may be responsible for a proportion of the genetically undiagnosed patients (28.82%). Additionally, the failure to identify mutations in these patients may be due to the causative variants being located in coding regions poorly covered by WES, in non-coding regions or regulatory sequences, or in novel genes that have not yet been associated with IRD. Interestingly, the percentage of unsolved patients was significantly higher in CD and CRD in comparison to other conditions (see Fig. 2c), suggesting that these dystrophies have been studied to a lesser extent, and that more novel genes are yet to be discovered for these particular phenotypes.

The phenotypic overlap of retinal dystrophies greatly diminishes the ability to arrive at an accurate clinical diagnosis. In fact, some authors estimate that IRD patients visit an average of seven ophthalmologists before the final diagnosis is made^40^. In our cohort, a significant group of patients (11.86%) showed clinical features that were compatible with different retinal dystrophies, prompting us to design a non-disease-specific panel that encompassed all syndromic and non-syndromic IRD genes. Once the analysis was performed, the pathogenic mutation was successfully identified in almost 72% of the families with an unclear phenotype, providing evidence that our strategy is effective not only for patients with a precise and particular clinical diagnosis, but also for cases with uncertain phenotypic features. Moreover, this non-hypothesis-driven approach allowed us to propose the already known IRD gene *CDHR1* as a good RP candidate. This gene was previously described in families mainly affected by CRD^41^,^42^,^43^, or by a retinal dystrophy that involves both rods and cones at the same time^22^,^44^. In our study, family Fi15/19 was found to carry mutations in *CDHR1*, and displayed the main clinical traits corresponding to autosomal recessive RP, with a remarkable premature degeneration of cones. These particular traits are similar to those observed in some patients that carry mutations in *PROM1*, a gene also involved in RP and CRD^45^. In fact, PROM1 and CDHR1 proteins co-localize at the base of the developing outer segment of photoreceptors, with both participating in disc morphogenesis^46^. This suggests that their alterations could lead to similar clinical phenotypes. Here, we propose *CDHR1* as a good candidate in molecular genetic studies of patients showing either CRD or RP symptoms, and add two new mutations to the molecular spectrum of this gene. Previously, only seven different *CDHR1* variants have been described, most of them resulting in premature stop codons or splicing alterations that should lead to NMD. In this work, we report for the first time *in vivo* functional assays for *CDHR1* mutations that demonstrate that pathogenic mechanisms other than NMD may be responsible for the deleterious effect of truncating/splicing variants.

In addition to basic molecular genotyping, in certain cases, functional studies are needed in order to provide a conclusive genetic diagnosis. This was the case for family Fi15/12, who carry an intronic deletion in *RPGRIP1* (c.2367 + 23del) that, according to *in silico* prediction programs, has a slight effect on splicing. However, here we provide *in vivo* functional evidence that this variant has a clear impact on the *RPGRIP1* splicing mechanisms. Similarly, a recent study demonstrated the pathogenic effect of a prevalent *ABCA4* intronic mutation, c.5461-10T > C, which was also predicted to be neutral by bioinformatic tools^47^. Together, these studies highlight the importance of functional analysis, with splicing predictors being frequently difficult to interpret and sometimes unreliable, especially for variants located outside of the intronic canonical signals. In addition, *in vivo* studies of mutations present in heterogeneous diseases, such as those investigated in the present analysis, are also useful for the establishment of genotype–phenotype correlations. This is the case for *CDH23*, where previous work has shown that truncated peptides or loss of numerous amino acid residues in the CDH23 protein results in US, whereas missense mutations cause non-syndromic deafness^48^,^49^. Such evidence is in accordance with the *in vivo* results that we obtained from the analysis of family Fi15/36, who is affected by US, and carries two new mutations altering the open reading frame of *CDH23*.

In conclusion, we analyzed a large cohort of clinically heterogeneous IRD patients using WES, and report a diagnostic yield greater than 70%. Altogether, our results indicate that WES using Ion Proton^TM^ system is a valuable strategy for application to IRD.

## Methods

### Patients

A cohort of 59 clinically and genetically unselected IRD families was included in the present study. None of the families was previously screened for gene mutations, except pedigree Fi15/12 that presented previous genetic data^23^, which was inconclusive and therefore not considered in this work. Most patients originated from Spain (n = 46), while the others were from the Arabian Peninsula (n = 11), Venezuela (n = 1), and Costa Rica (n = 1). Clinical diagnoses were established at the Institut de Microcirurgia Ocular (Barcelona, Spain) and were based on standard ophthalmic evaluations (best corrected visual acuity, retinography, fundus autofluorescence, optical coherence tomography, electroretinography, and visual field). Peripheral blood (in EDTA tubes) or saliva samples were obtained from patients and their relatives. Automated extraction of genomic DNA was performed by using the KingFisher Duo purification system (Thermo Fisher Scientific, Waltham, MA). All procedures used in this study were in accordance with the Declaration of Helsinki. Ethics approval was received from the Ethics Committee of Institut de Microcirurgia Ocular (160321_96). All patients and their relatives were fully informed of the purpose and procedures of this study, and written consent was obtained from each individual.

### Gene panel design, whole exome sequencing and data processing

A total of 212 genes previously associated with inherited retinal disorders were included in our IRD panel. Genes were selected according to the information available in RetNet (https://sph.uth.edu/retnet/) and Pubmed databases (http://www.ncbi.nlm.nih.gov/pubmed/). These genes were known to be responsible for 13 non-syndromic IRD forms (CD, CRD, LCA, RP, ACHR, STGD, congenital stationary night blindness, gyrate atrophy, familial exudative vitreoretinopathy, choroideremia, Sorsby’s dystrophy, Norrie disease, and retinoschisis) and more than 10 syndromic diseases (US, BBS, JS, Alström syndrome, Senior–Løken syndrome, Stickler syndrome, Alport syndrome, Alagille syndrome, Wagner disease, and oculoauricular syndrome, among others).

WES was performed in patients from 59 unrelated families using libraries designed and constructed using the Ion AmpliSeq^TM^ Exome technology (ThermoFisher Scientific). Generated amplicons were genotyped with the Ion Proton^TM^ platform (Life Technologies), following the manufacturer’s instructions. Sequences were aligned against the reference genome (GRCh37/hg19) by using TMAP Alignment (Thermo Fisher Scientific). WES was performed in collaboration with a private company (NIMGenetics, Madrid, Spain), which provided the BAM, BAI, and FASTQ files, as well as VCF and TSV files that contained a compilation of all of the variants detected using the Ion Reporter software (Thermo Fisher Scientific). Moreover, the company supplied a TSV file that specifically included the variants identified in the 212 genes (after running a custom pipeline), and a document that detailed the coverage data of the panel, specifying the number of reads of each amplicon. The coverage information obtained from the 59 samples was used to measure the average depth and coverage percentage of each gene.

### Determination of pathogenic variants

Variants that were detected in genes included in the IRD panel were filtered according to coverage (≥15x), minor allele frequency (≤0.01), and deleterious potential. All resulting variants were contrasted with the mutation databases, HGMD (http://www.hgmd.cf.ac.uk/ac/index.php) and Uniprot (http://www.uniprot.org/). The pathogenicity of missense changes was evaluated using the following *in silico* predictors: SIFT, MutationTaster, PolyPhen-2, and Align GV-GD. When new variants potentially affected the splicing mechanism, the splice site score values of the wild-type and the mutated sequence were predicted online using SpliceSite, MaxEntScan, NNSPLICE, GeneSplicer and Human Splicing Finder. Moreover, nucleotide conservation was evaluated using the PhastCons and PhyloP programs.

Sanger sequencing was performed to confirm all of the putative pathogenic variants obtained after WES genotyping, and mutation segregation analysis was carried out when relatives’ samples were available.

In partially solved recessive cases, with only one pathogenic variant detected in an IRD gene, the coverage data of this gene was carefully evaluated, and those regions poorly covered (<15x) were Sanger sequenced. If the second pathogenic allele was not found, and gross deletion or duplication rearrangements were previously described in the gene of interest, a copy number variation analysis was also carried out. In particular, in family Fi15/40, a deletion/duplication analysis for *GPR98* was performed using a custom designed gene centric microarray (Baylor Miraca, Houston, TX). In family Fi15/44 Supplementary Table S4, multiplex ligation-dependent probe amplification (MLPA) analysis was used to evaluate the presence of rearrangements of *USH2A* (SALSA MLPA probemixes P361 and P362).

### RNA expression

Total RNA from particular patients and their relatives was obtained from 500 μl of blood stabilized with 1.3 ml of RNAlater by using the RiboPure-Blood purification kit (Thermo Fisher Scientific), according to the manufacturer’s instructions. First, cDNA chains were obtained by reverse transcription (RT) using the Transcriptor High Fidelity cDNA Synthesis Kit (Roche Diagnostics, Indianapolis, IN). Specific amplification of transcripts was obtained for *MERTK, RPGRIP1, CDH23* or *CDHR1* genes. *GAPDH* was used as a control for normalization. Primer sequences are given in Supplementary Table S5.

Quantitative real-time RT-PCR was performed using Taqman^TM^ Gene Expression Assays (Applied Biosystems, Carlsbad, CA) from 20–30 ng of cDNA per well. Relative gene expression was assayed in triplicate and compared with wild-type samples, which served as the set point. All real-time RT-PCR reactions were performed on a QuantStudio 3 instrument (Applied Biosystems), following the manufacturer’s instructions. Housekeeping genes β*2 M, GAPDH* and *ACTB* were used for normalization. Relative quantification was assessed by using the 2^−ΔΔCT^ method. Taqman Gene Expression Assays references were Hs00187842_m1 (β*2 M*), Hs99999905_m1 (*GAPDH*), Hs01060665_g1 (*ACTB*), Hs01031970_m1 (*MERTK*) and Hs00971456_g1 (*RPGRIP1*).

## Additional Information

**How to cite this article**: Riera, M. *et al*. Whole exome sequencing using Ion Proton system enables reliable genetic diagnosis of inherited retinal dystrophies. *Sci. Rep.* **7**, 42078; doi: 10.1038/srep42078 (2017).

**Publisher's note:** Springer Nature remains neutral with regard to jurisdictional claims in published maps and institutional affiliations.

## Supplementary Material

Supplementary Information

## Figures and Tables

**Table 1:** Clinical classification of the families included in our cohort.

Disease	Families	Percentage
Retinitis pigmentosa	26	44.06%
Usher syndrome	6	10.16%
Cone dystrophy	6	10.16%
Stargardt disease	5	8.47%
Leber congenital amaurosis	4	6.77%
Cone-rod dystrophy	3	5.08%
Achromatopsia	1	1.69%
Gyrate atrophy	1	1.69%
Unclear phenotype	7	11.86%
Total	59	100%

**Table 2:** Overview of the variants obtained in the genetically solved cases of our cohort.

Family ID	Phenotype	Gene	Allele 1		Reference	Allele 2		Reference	Segregation
			Nucleotide change	Protein change		Nucleotide change	Protein change		
**Autosomal dominant cases** (**6/42**, **14.28%**)									
Fi15/01*	CD	GUCY2D	c.2513G > A	Arg838His	50				Yes
Fi15/02*	CD	GUCY2D	c.2512C > T	Arg838Cys	51				Yes
Fi15/03*	MD vs SFD	TIMP3	c.499G > A	Asp167Asn	This study				Yes
Fi15/04*	LCA	CRX	c.785del	Ser262Thrfs*109	This study				Yes
Fi15/05*	RP	PRPF8	c.6926A > G	His2309Arg	52				Yes
Fi15/06*	RP	RHO	c.872C > G	Pro291Arg	This study				No
**Autosomal recessive cases** (**34/42**, **80.95%**)									
Fi15/07	ACHR	CNGA3	c.847C > T	Arg283Trp	53	c.847C > T	Arg283Trp	53	No
Fi15/08	ACHR vs CD	PDE6C	c.1574G > T	Gly525Val	This study	c.1574G > T	Gly525Val	This study	Yes
Fi15/09	CRD	PROM1	c.3G > A	Met1Ile	This study	c.1354_1355insT	Tyr452Leufs*13	54	Yes
Fi15/10	GA	OAT	c.627T > A	Tyr209Ter	55	c.627T > A	Tyr209Ter	55	Yes
Fi15/11	LCA	CEP290	c.1864_1865del	Asp622Phefs*5	This study	c.4723A > T	Lys1575Ter	56	Yes
Fi15/12	LCA	RPGRIP1	c.895_896del	Glu299Serfs*21	23	c.2367 + 23del	Splicing	23	Yes
Fi15/13	LCA	CRB1	c.611_617del	Ile205Aspfs*13	57	c.2843G > A	Cys948Tyr	58	Yes
Fi15/14	LCA vs CD vs RP	CNGB3	c.1148del	Thr383fs	59	c.1148del	Thr383fs	59	Yes
Fi15/15	RP	PDE6A	c.1630C > T	Arg544Trp	60	c.1630C > T	Arg544Trp	60	Yes
Fi15/16	RP	EYS	c.4120C > T	Arg1374Ter	61	c.4829_4832del	Ser1610Phefs*7	This study	Yes
Fi15/17	RP	PDE6A	c.1630C > T	Arg544Trp	60	c.1630C > T	Arg544Trp	60	Yes
Fi15/18	RP	USH2A	c.2633G > A	Arg878His	62	c.11927C > T	Thr3976Met	63	Yes
Fi15/19*	RP	CDHR1	c.1485 + 2T > C/c.1554-2A > C	Splicing	22/This study	c.1868_1869insA	Asn623Lysfs*53	This study	Yes
Fi15/20*	RP	MERTK	c.1961G > T	Gly654Val	This study	c.1961G > T	Gly654Val	This study	Yes
Fi15/21*	RP	USH2A	c.12574C > T	Arg4192Cys	64	c.12574C > T	Arg4192Cys	64	Yes
Fi15/22	RP	PDE6B	c.299G > A	Arg100His	38	c.299G > A	Arg100His	38	Yes
Fi15/23*	RP	ABCA4	c.1804C > T	Arg602Trp	65	c.5819T > C	Leu1940Pro	66	Yes
Fi15/24*	RP	PRCD	c.70C > T	Gln24Ter	This study	c.70C > T	Gln24Ter	This study	Yes
Fi15/25	RP	RPE65	c.292_311del	Ile98Hisfs*26	67	c.419G > A	Gly140Glu	This study	Yes
Fi15/26*	RP	PDE6B	c.1860del	His620Glnfs*23	68	c.1860del	His620Glnfs*23	68	No
Fi15/27	RP	EYS	c.6111C > A	Cys2037Ter	This study	c.6111C > A	Cys2037Ter	This study	No
Fi15/28	RP	PDE6A	c.305G > A	Arg102His	69	c.1268del	Leu423Ter	This study	Yes
Fi15/29*	RP vs CRD	CRB1	c.498_506del	Ile167_Gly169del	70	c.2843G > A	Cys948Tyr	58	Yes
Fi15/30	ARB vs STGD	BEST1	c.798del	Gln327Argfs*42	This study	c.798del	Gln327Argfs*42	This study	Yes
Fi15/31*	STGD	ABCA4	c.5461-1G > T	Splicing	This study	c.6118C > T	Arg2040Ter	71	Yes
Fi15/32	STGD	ABCA4	c.514G > A, c.2023G > A, c.6148G > C	Gly172Ser, Val675Ile, Val2050Leu	72, 73, 74	c.3211_3212insGT	Ser1071fs*14	74	Yes
Fi15/33*	STGD	ABCA4	c.3988G > T	Glu1330Ter	64	c.5882G > A	Gly1961Glu	74	Yes
Fi15/34	STGD	ABCA4	c.2041C > T	Arg681Ter	75	c.4919G > A	Arg1640Gln	76	Yes
Fi15/35*	US	MYO7A	c.3719G > A	Arg1240Gln	77	c.5886_5888del	Phe1963del	78	Yes
Fi15/36	US	CDH23	c.5546del	Pro1849Leufs*4	This study	c.7482 + 1G > A	Splicing	This study	Yes
Fi15/37	US	USH2A	c.10636G > A	Gly3546Arg	79	c.10636G > A	Gly3546Arg	79	Yes
Fi15/38	US	USH2A	c.9799T > C	Cys3267Arg	80	c.9799T > C	Cys3267Arg	80	Yes
Fi15/39*	US	CLRN1	c.254-1G > A	Splicing	This study	c.254-1G > A	Splicing	This study	Yes
Fi15/40	US	GPR98	c.7988_7989del	Ser2663Ter	This study	exon 2–50 deletion	Gross deletion	This study	Yes
**X-linked cases** (**2/42**, **4.76%**)									
Fi15/41*	RP	RPGR	c.2235_2236del	Glu746Argfs*23	81				Yes
Fi15/42	RP	RP2	c.358C > T	Arg120Ter	82				Yes

Asterisks (^*^) highlight those families with more than one affected member, commas (,) separate variants in the same allele, and slashes (/) depict cases where different mutations were identified in different affected members of the same family. The number of genetically diagnosed families is shown for each inheritance, and the percentage of each pattern is calculated on the solved cases (42 in total). Abbreviations: ACHR, achromatopsia; ARB, autosomal recessive bestrophinopathy; CD, cone dystrophy; CRD, cone–rod dystrophy; GA, gyrate atrophy; LCA, Leber congenital amaurosis; MD, macular dystrophy; RP, retinitis pigmentosa; SFD, Sorsby’s fundus dystrophy; STGD, Stargardt disease; US, Usher syndrome.

**Table 3:** Comparison of mutation detection rate and coverage data of different studies that have been used next-generation sequencing for the genetic analysis of an IRD cohort.

Reference	Mutation detection rate	Num. of patients	Patient’s phenotype	Genes included	Method	Sequencing platform	Mean depth	% bp > 10X	% bp > 20X
**Whole exome sequencing**									
This study	71.1%	59	IRD	212 IRD genes	Ion AmpliSeq^TM^ Exome (Life Technologies)	Ion Proton	121×	97.5%	96.9%
Tiwari *et al*.^83^	64%	58	IRD	250 IRD genes	SeqCap EZ NimbleGen (Roche)/ Nextera Rapid Capture Exome (Illumina)	Illumina HiSeq 2000/Illumina NextSeq500	—	—	—
Xin *et al*.^29^	57.6%	33	STGD	163 IRD genes	SureSelect v4 (Agilent Technologies)	Illumina HiSeq 2000	125×	—	—
Beryozkin *et al*.^30^	48.5%	68	IRD	226 IRD genes	SeqCap EZ NimbleGen (Roche)	Illumina HiSeq 2000	80×	—	—
**Targeted next-generation sequencing**									
Bravo-Gil *et al*.^31^	73%	32	IRD	64 IRD genes	Custom SureSelect (Agilent Technologies)	Illumina MiSeq	409×	—	—
Eisenberg *et al*.^11^	70%	126	RP and LCA	55 RP and LCA genes	SeqCap EZ NimbleGen (Roche)	Roche GS FLX/ Illumina MiSeq	75× 250×	90.0% 99.0%	—
Aparisi *et al*.^10^	68.7%	44	US	14 US genes	HaloPlex (Agilent Technologies)	Illumina MiSeq	1334×	—	—
Patel *et al*.^32^	62.3%	292	IRD	322 IRD genes	Ion Ampliseq custom panel (Life Technologies)	Ion Proton	—	—	—
Boulanger-Scemama *et al*.^19^	62.1%	95	CD and CRD	123 IRD genes	Custom SureSelect (Agilent Technologies)	Illumina Genome Analyzer	244×	—	—
Zhao *et al*.^33^	60%	82	RP	186 IRD genes	Ion Ampliseq custom panel (Life Technologies)	Illumina HiSeq 2000	—	95.1%	—
Perez-Carro *et al*. (2015)^9^	57.4%	47	RP	75 RP genes	HaloPlex (Agilent Technologies)	Illumina MiSeq	722×	99.1%	—
Huang *et al*.^34^	55.3%	179	IRD	164 IRD genes	GenCap (MyGenostics)	Illumina HiSeq 2000	191×	98.2%	—
Glöckle *et al*.^13^	55–80%	170	IRD	105 IRD genes	Custom SureSelect (Agilent Technologies)	SOLiD	750×	—	—
O’Sullivan *et al*.^35^	50–55%	50	RP	105 IRD genes	Custom SureSelect (Agilent Technologies)	SOLiD	**—**	**—**	92.0%
Weisschuh *et al*.^36^	50%	50	IRD	105 IRD genes	Custom SureSelect (Agilent Technologies)	SOLiD	750×	**—**	**—**
Oishi *et al*.^37^	36.3–50%	329	RP and US	193 IRD genes	HaloPlex (Agilent Technologies)	Illumina HiSeq 2500	250×	92.2%	88.7%
Neveling *et al*.^38^	36%	100	RP	111 IRD genes	12-plex NimbleGen (Roche)	Roche GS FLX	—	89.0%	—
Shanks *et al*.^39^	25%	36	IRD	73 IRD genes	12-plex NimbleGen (Roche)	Roche 454	—	95.0%	85.0%

Studies are classified according to the method and sorted by descending order of mutation detection rate. Only those studies that included a minimum of 30 patients are mentioned. Abbreviations: CD, cone dystrophy; CRD, cone-rod dystrophy; IRD, inherited retinal dystrophies; LCA, Leber congenital amaurosis; RP, retinitis pigmentosa; STGD, Stargardt disease; US, Usher syndrome.
