# Supplementary material for: Whole exome sequencing using Ion Proton system enables reliable genetic diagnosis of inherited retinal dystrophies
Source: Sci Rep. 2017 Feb 9;7:42078. doi: 10.1038/srep42078 (PMC5299602; doi:10.1038/srep42078)
Supplement: Supplementary Information [file srep42078-s1.pdf]

Whole exome sequencing using Ion Proton system enables reliable genetic diagnosis of inherited retinal dystrophies

Marina Riera, Rafael Navarro, Sheila Ruiz-Nogales, Pilar Méndez, Anniken Burés-Jelstrup, Borja Corcóstegui, Esther Pomares

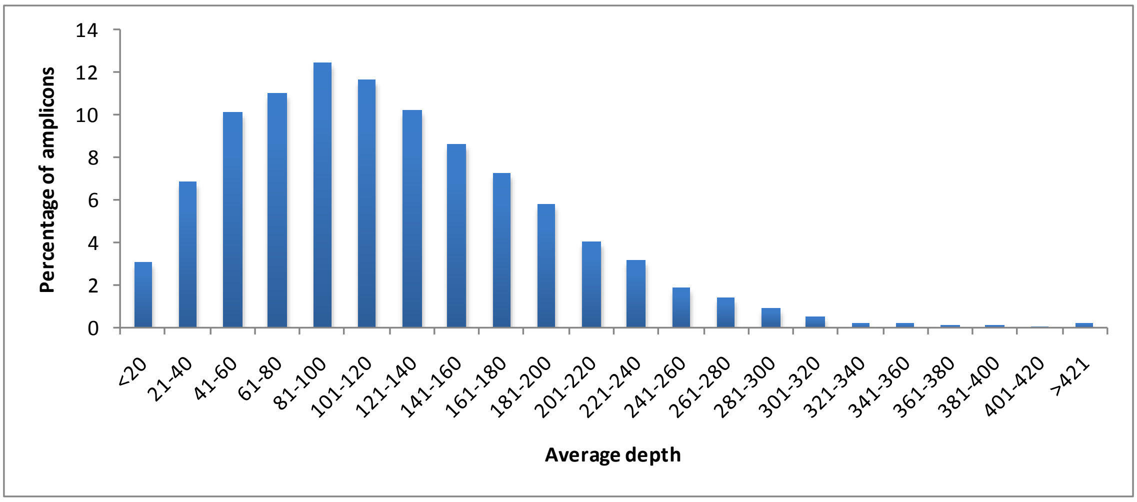

Supplementary figure S1. Percentage of targeted amplicons covered at different depth ranges. Each bar represents the average depth score obtained from the 59 samples.

Supplementary table S1. List of the 212 genes included in the IRD panel.

ABCA4	*CRX*	*LRP5*	*RBP3/IRBP*
ABHD12	*CSPP1*	*LZTFL1*	*RBP4*
ADAM9	*CX3CR1*	*MAK*	*RD3*
AIPL1	*CYP4V2*	*MERTK*	*RDH11*
ALDH18A1	*DFNB31*	*MFN2*	*RDH12*
ALMS1	*DHDDS*	*MFRP*	*RDH5*
ARL2BP	*DSPP*	*MFSD8*	*RGR*
ARL6	*DTHD1*	*MKKS*	*RGS9*
ARMS2	*EFEMP1*	*MKS1*	*RGS9BP*
BBS1	*ELOVL4*	*MYO7A*	*RHBDD2*
BBS10	*ERCC6*	*NDP*	*RHO*
BBS12	*EYS*	*NEK2*	*RIMS1*
BBS2	*FAM161A*	*NEUROD1*	*CRALBP*
BBS4	*FBLN5*	*NMNAT1*	*ROM1*
BBS5	*FLVCR1*	*NPHP1*	*RP1*
BBS7	*FSCN2*	*NPHP3*	*RP1L1*
BBS9	*FZD4*	*NPHP4*	*RP2*
BEST1	*GDF6*	*NR2E3*	*RP9*
C1QTNF5	*GIPC3*	*NR2F1*	*RPE65*
C2	*GNAT1*	*NRL*	*RPGR*
C2ORF71	*GNAT2*	*NYX*	*RPGRIP1*
C3	*GNPTG*	*OAT*	*RS1*
C8ORF37	*GPR179*	*OFD1*	*SAG*
C9	*GPR98*	*OPA1*	*SDCCAG8*
CA4	*GRK1*	*OPA3*	*SEMA4A*
CABP4	*GRM6*	*OPN1LW*	*SLC24A1*
CACNA1F	*GUCA1A*	*OPN1MW*	*SNRNP200*
CACNA2D4	*GUCA1B*	*OPN1SW*	*SPATA7*
CC2D2A	*GUCY2D*	*OR2W3*	*TEAD1*
CCDC28B	*HARS*	*OTX2*	*TIMM8A*
CDH23	*HGSNAT*	*PANK2*	*TIMP3*
CDH3	*HK1*	*PCDH15*	*TLR3*
CDHR1	*HMCN1*	*PDE6A*	*TMEM126A*
CEP164	*HMX1*	*PDE6B*	*TMEM237*
CEP290	*HTRA1*	*PDE6C*	*TNC*
CERKL	*IDH3B*	*PDE6G*	*TOPORS*
CFB	*IMPDH1*	*PDE6H*	*TRIM32*
CFH	*IMPG1*	*PDZD7*	*TRPM1*
CFI	*IMPG2*	*PGK1*	*TSPAN12*
CHM	*INVS*	*PITPNM3*	*TTC8*
CIB2	*IQCB1*	*PNPLA6*	*TTLL5*
CLN3	*ITM2B*	*POC1B*	*TTPA*
CLRN1	*JAG1*	*PRCD*	*TUB*
CNGA1	*KCNJ13*	*PRKCG*	*TULP1*
CNGA3	*KCNV2*	*PROM1*	*UNC119*
CNGB1	*KIAA1549*	*PRPF3*	*USH1C*
CNGB3	*KIZ*	*PRPF31*	*USH1G*
CNNM4	*KLHL7*	*PRPF6*	*USH2A*
COL11A1	*LCA5*	*PRPF8*	*VCAN*
COL2A1	*LHFPL5*	*PRPH2/RDS*	*WDPCP*
COL4A6	*LOXHD1*	*RAB28*	*WDR19*
COL9A1	*LRAT*	*RAX2*	*ZNF408*
CRB1	*LRIT3*	*RB1*	*ZNF513*

Supplementary table S2. Pathogenicity predictions for new missense mutations.

Family ID	Gene	Nucleotide change	Protein change	PhastCons	PhyloP	SIFT	Mutation Taster	PolyPhen2	Align GVGD	ExAC	ESP	Reference
Fi15/03	TIMP3	c.499G>A	Asp167Asn	1	5,53	Tolerated (0,72)	Disease causing (23)	Possibly damaging (0,91)	C0	-	-	This study
Fi15/06	RHO	c.872C>G	Pro291Arg	1	6,1	Deleterious (0)	Disease causing (103)	Probably damaging (1)	C65	-	-	This study
Fi15/08	PDE6C	c.1574G>T	Gly525Val	1	5,45	Deleterious (0)	Disease causing (109)	Possibly damaging (1)	C65	-	-	This study
Fi15/20	MERTK	c.1961G>T	Gly654Val	1	5,86	Deleterious (0)	Disease causing (109)	Probably damaging (1)	C0	-	-	This study
Fi15/25	RPE65	c.419G>A	Gly140Glu	1	5,94	Deleterious (0,02)	Disease causing (98)	Possibly damaging (0,94)	C0	-	-	This study
Fi15/44	USH2A	c.8767G>A	Gly2923Ser	0,69	2,79	Deleterious (0)	Disease causing (56)	Probably damaging (1)	C55	-	-	This study

Supplementary table S3. Pathogenicity predictions for new splicing mutations.

Family ID	Gene	Nucleotide change	Nucleotide Conservation		Splicesite		MaxEntScan		NNSPLICE		GeneSplicer		HSF		ExAc	ESP	Reference
			PhastCons	PhyloP	WT	MUT	WT	MUT	WT	MUT	WT	MUT	WT	MUT			
Fi15/12	RPGRIP1	c.2367+23del	0	0,29	87,25	87,25	7,66	7,66	0,93	0,93	4,17	3,86	94,74	94,74	0,000083	0,0005	de Castro Miro 2014
Fi15/19	CDHR1	c.1554-2A>C	1	3,11	76,68	-	8,9	-	-	-	4,21	-	85,99	-	-	-	This study
Fi15/20	MERTK	c.1961G>T	1	5,86	85,46	-	6,99	-	0,94	-	-	-	90,04	-	-	-	This study
Fi15/31	ABCA4	c.5461-1G>T	1	5,77	89,32	-	10,03	-	0,89	-	10,47	-	88,45	-	-	-	This study
Fi15/36	CDH23	c.7482+1G>A	1	6,1	90,62	-	10,07	-	0,91	-	9,21	-	96,91	-	-	-	This study
Fi15/39	CLRN1	c.254-1G>A	1	5,05	86,96	-	5,98	-	0,86	-	6,15	-	90,17	-	-	-	This study

Supplementary table S4. Genetically unsolved cases of our cohort.

Patient ID	Phenotype	Gene	Allele 1		Reference
			Nucleotide change	Protein change	
**Cases with only one pathogenic variant in a recessive gene**					
Fi15-43*	STGD	ABCA4	c.3988G>T	Glu1330Ter	Corton et al. 2013
Fi15-44	RP	USH2A	c.8767G>A	Gly2923Ser	This study
Fi15-45	CD	ABCA4	c.2701A>G	Thr901Ala	Rivera et al. 2000
Fi15-46*	CRD	CRB1	c.614T>C	Ile205Thr	Bernal et al. 2003
**No pathogenic variant detected**					
Fi15-47	CD	-			
Fi15-48*	CD	-			
Fi15-49*	CD	-			
Fi15-50	RP	-			
Fi15-51*	RP	-			
Fi15-52*	RP	-			
Fi15-53	RP	-			
Fi15-54*	RP	-			
Fi15-55	RP	-			
Fi15-56	RP	-			
Fi15-57	CRD	-			
Fi15-58	RP vs US	-			
Fi15-59*	STGD vs CACD	-			

Asterisks (*) highlight those families with more than one affected member.
Abbreviations: CACD, central areolar choroidal dystrophy; CD, cone dystrophy; CRD, cone-rod dystrophy; MD, macular dystrophy; RP, retinitis pigmentosa; STGD, Stargardt disease; US, Usher syndrome.

Supplementary table S5. Sequences of primers used in RT-PCR reactions.

Gene	Primer sequence (5' → 3')	
	**Forward**	**Reverse**
GAPDH	TGAAGGTCGGAGTCAACGGATTTGG	CATGTAGGCCATGAGGTCCACCAC
MERTK	ATGCATTCACAGAGGAGGATTC	CTGCTTCAACCTGTGGCCATG
CDH23	ACACGGAAGGTGAACATCCAG	TAGACCTCGTACACGTTGGAG
RPGRIP1	TCCATGGCTTGGCCACACTG	CTTTGAACTGTCCTTGGTATCC
CDHR1	AGCCATCTCCATCACTCAGAG	GATGGAATAGTCCACCAGGTTG
